# Accurate and precise real-time RT-PCR assays for the identification of astrovirus associated encephalitis in cattle

**DOI:** 10.1038/s41598-018-27533-8

**Published:** 2018-06-15

**Authors:** Ramona Lüthi, Céline L. Boujon, Ronja Kauer, Michel C. Koch, Ilias G. Bouzalas, Torsten Seuberlich

**Affiliations:** 1 NeuroCenter, Division of Neurological Sciences, Vetsuisse Faculty, University of Bern, Bern, Switzerland; 2 Graduate School for Cellular and Biomedical Sciences, University of Bern, Bern, Switzerland; 3 Present Address: Veterinary Research Institute, Hellenic Agricultural Organization-DEMETER, Campus of Thermi, Thessaloniki, Greece

## Abstract

A novel bovine astrovirus genotype species (BoAstV-CH13/NeuroS1) was recently identified in brain tissues of cattle as a plausible cause of encephalitis. The purpose of the present study was to develop and validate real time RT-PCR assays for the detection of BoAstV-CH13/NeuroS1 in brain tissues of cattle. Three different primer-probe combinations were designed based on BoAstV-CH13/NeuroS1 full-genome sequences of 11 different strains identified in cattle, and established in three distinct one-step real time RT-PCR protocols. These protocols were compared regarding their diagnostic performance using brain tissues of cattle with and without astrovirus associated encephalitis. The limit of detection (LOD) of all three assays was between 1.34 × 10^1^ and 1.34 × 10^2^ RNA copies, leading to an analytical sensitivity two orders of magnitude superior compared to a conventional pan-astrovirus RT-PCR protocol (LOD 1.31 × 10^4^ RNA copies). Amplification efficiency was in the range of 97.3% to 107.5% with linearity (R^2^) > 0.99. The diagnostic sensitivity and specificity of the assays was determined as 100%, and all three revealed good intra- and inter-test repeatability. In conclusion, the newly developed RT-qPCRs are sensitive, specific, and reliable test formats that will facilitate BoAstV-CH13/NeuroS1 detection in routine diagnostics as well as in research settings.

## Introduction

*Astroviridae* are non-enveloped positive-sense single-stranded RNA viruses of a broad genetic diversity causing infections in mammalian and avian species. In infants, infections with classical human astroviruses are among the main causes of diarrhea; however, intestinal astrovirus infections in mammalian animal species oftentimes remain subclinical and do not cause pathological changes in the host^1^. Using broadly reactive pan-astrovirus RT-PCR protocols and next–generation sequencing-based viral metagenomics, the spectrum of known astrovirus genotype species and related pathologies has remarkably expanded over the past decade^2^. Novel astroviruses have been found in association with encephalitis and neurological disease in mink^3^, humans^4^, cattle^5–8^ sheep^9,10^ and pigs^11–13^. Most of these encephalitis-associated strains are phylogenetically related, belong to the so-called human-mink-ovine (HMO)-like astrovirus cluster and show less than 50% sequence similarity of structural and nonstructural viral proteins compared to the classical, gut-associated astrovirus strains^2^.

In cattle, bovine astrovirusCH13/NeuroS1 (BoAstV-CH13/NeuroS1) was detected in brain tissues of up to 40% of animals with non-suppurative encephalitis of previously unresolved etiology in Europe and North America^6,14–17^. Viral RNA and proteins were consistently detected in the neurons in affected brain tissues in association with pathological lesions, such as perivascular cuffs, neuronal necrosis and gliosis, which supports a plausible causal relationship of astrovirus infection and encephalitis^14,18^. However, more comprehensive epidemiological data and pathogenesis studies are still missing.

Current techniques for BoAstV-CH13/NeuroS1 detection in brain tissues either rely on *in* *situ* detection of viral RNA, or viral proteins in formalin-fixed and paraffin-embedded tissue sections or on conventional or nested RT-PCR procedures^5,6,10,18^. As the virus has not been isolated in cell culture so far, *in* *situ* techniques are important tools to investigate and characterize pathological samples, but these procedures are rather laborious and time-consuming, and require dedicated technical equipment as well as specifically trained personnel. Conventional RT-PCR protocols are sensitive, but prone to contaminations and results may be biased by RNA extraction failure and/or RNA degradation. In consequence, there is a need for rapid, sensitive, specific and reliable molecular diagnostic procedures that can be applied in routine diagnostics as well as in research settings.

The aim of the present study was to develop and validate quantitative fluorescent reporter-probe based real-time RT-PCR protocols for the purpose of post-mortem diagnosis of BoAstV-CH13/NeuroS1 infection in brain tissues of neurologically diseased cattle. Three different assays that target different regions of the viral genome performed almost equally well in terms of their analytical sensitivity, diagnostic sensitivity, diagnostic specificity and repeatability and thus represent valuable extensions of the diagnostic toolbox available for BoAstV-CH13/NeuroS1.

## Materials and Methods

### Tissue samples

BoAstV-CH13/NeuroS1 positive brain samples (n = 20) derived from neurologically diseased cattle, whose brains were submitted post-mortem to the diagnostic service of the NeuroCenter, Division of Neurological Sciences, Vetsuisse Faculty, University of Bern for neuropathological examination. All animals showed histopathologically moderate or severe non-suppurative encephalitis and were positive for BoAstV-CH13/NeuroS1 by a conventional pan-astrovirus RT-PCR^19^, by *in* *situ* hybridization^5^ or by immunohistochemistry^18^. Details on these animals are provided in Table 1. There was no evidence that individual animals were geographically related or in contact to each other. Negative control samples consisted of medulla oblongata tissues that were tested negative for BoAstV-CH13/NeuroS1 in the pan-astrovirus RT-PCR and by next-generation RNA sequencing^17^. According to their origin and their histopathology pattern, negative control samples were categorized into two groups: (i) samples derived from regular slaughtered, healthy adult cattle that did not reveal histopathological lesions (n = 20) and (ii) samples of fallen cattle with histopathological lesions of a non-suppurative inflammatory pattern in the medulla oblongata (n = 20).

**Table 1:** Details on bovine astrovirus-CH13/NeuroS1 positive animals and brain tissues used in the present study.

Animal ID	Age [years]	Morphological diagnosis	Virus characterisation methods^a^	References	Tissues used in this study
23985	2.5	Severe non-suppurative meningoencephalitis	RT-PCR, ISH, NGS, IHC	^5,18,21^	cerebral cortex
26730	4	Viral meningoencephalitis	ISH, NGS, IHC	^18,21^	cerebral cortex, cerebellum
26875	1.5	Viral meningoencephalitis	RT-PCR, ISH, NGS, IHC	^5,21^	brainstem
27020	3	Viral meningoencephalitis	ISH, IHC	^18^	cerebral cortex, cerebellum
36716,	2	Non-suppurative meningo-encephalopmyelitis	ISH, NGS, IHC	^18,21^	cerebral cortex, cerebellum, brainstem
37514	2.5	Moderate non-suppurative meningo-encephalopmyelitis	RT-PCR, ISH, IHC	^5,14^	cerebral cortex
42268	4	Moderate non-suppurative meningo-encephalopmyelitis	ISH, IHC	^14^	cerebral cortex, cerebellum, brainstem
42799	6.5	Severe non-suppurative polioencephalomyelitis	RT-PCR, ISH, NGS, IHC	^5,8,14,21^	brainstem
43660	4.5	Moderate non-suppurative meningo-encephalitis	ISH, NGS	^14,21^	brainstem
45664	1.5	Severe meningoencephalomyelitis	RT-PCR, ISH, IHC	^5,18^	cerebral cortex, cerebellum
49852	4.5	Severe non-suppurative encephalitis	RT-PCR, ISH, IHC	^14,24^	brainstem
50773	3.5	Severe non-suppurative poliomeningoencephalitis	RT-PCR, ISH, IHC	^18,21^	cerebral cortex, cerebellum

^a^RT-PCR, reverse-transcription polymerase chain reaction; ISH, *in* *situ* hybridization; IHC, immunohistochemistry; NGS, next-generation sequencing.

### RNA extracts

Brain tissue RNA extracts of cattle with non-suppurative encephalitis and a positive RT-PCR/PCR for bovine viral diarrhea virus (BVDV), ovine herpesvirus 2 (OvHV-2), bovine retrovirus CH15 (BoRV CH15), bovine polyomavirus 2 (BoPyV-2), bovine herpesvirus 6 (BHV-6), and/or parainfluenzavirus 5 (PIV-5) (n = 17) were available from the archives of the NeuroCenter (Table S1).

Feces samples of cattle (n = 148) were collected from the routine parasitology diagnostic service at the University of Bern. RNA was extracted using the QIAamp Viral RNA Mini kit (Qiagen) and screened for gastro-intestinal bovine astrovirus strains by conventional pan-astrovirus RT-PCR, Sanger sequencing and sequence comparison with the NCBI nucleotide database (Geneious software). We identified in total 6 samples with high sequence similarities to different astrovirus genotype species that had previously been detected in bovine feces (Table S2). In addition, eight pan-astrovirus RT-PCR negative feces samples served as controls.

### Astrovirus primer and probe design

Primers and probes for the BoAstV-CH13/NeuroS1 RT-qPCRs were designed based on the alignment of full-genome sequences of 11 different isolates from Switzerland and the USA using the Geneious Software package (Biomatters, version 9.1.8) and the Primer3 plugin (version 2.3.4; http://primer3.sourceforge.net/). Three different primer-probe combinations,  targeting different regions of the BoAstV-CH13/NeuroS1 genome, were selected: the 5′ end of the open-reading frame (ORF) 1a, the center of the genome at the ORF1a-ORF1b interception, and the 3′ end of ORF2. The respective protocols were designated RT-qPCR CH13-A, -B and -C. Details on all oligonucleotides are provided in Fig. S1 and Table 2. Cross reactivity of the oligonucleotides was checked by BLASTn and none of them, except one, revealed a similarity >80% with astrovirus sequences other than BoAstV-CH13/NeuroS1. Only the probe BAstV-qP of the CH13-B protocol matched also the human astrovirus BF34 (KF859964.1), the human astrovirus VA5 (KJ656124.1) and a bat astrovirus (FJ571067.1).

**Table 2:** Oigonucleotide sequences.

RT-qPCR (amplicon size)	Oligonucleotide name	Primer/probe sequence (binding sites in reference genome)^a,b^
CH13-A (208 bp)	CH13_488Fq	5′-AGGCATGACTATGAGCGCGT-3′ (488–507)
	CH13_609Pq	5′-^FAM^GGCAACGCACAGGCACTTG^BHQ1^-3′ (609–627)
	CH13_695Rq	5′-AATCCGGTTGTGCCACCTCA-3′ (695–676)
CH13-B (167 bp)	BAstV_qL	5′-TTTTGGCTCGTCACTTTGTG-3′ (4058–4077)
	BAstV_qP	5′-^FAM^GATAAGCTTTGGAGGGGAGG-^BHQ1^-3′ (4123–4142)
	BAstV_qR	5′-ACAACCTCCTTGGCAATCTG-3′ (4224–4205)
CH13-C (112 bp)	CH13_6339Fq	5′-GCACTCCCTTGCAGCAAGTC-3′ (6339–6358)
	CH13_6382Pq	5′-^FAM^CACCCACGCAGAAGCAGTTG^BHQ1^-3′ (6382–6401)
	CH13_6450Rq	5′-CTCGATCCTACTCGGCGTGG-3′ (6450–6430)
CDV (290 bp)	CDV_N-Fq	5′-ACT ATT GAA TCC CTT ATG ATG CTA-3′ (990–1013)
	CDV_N-Pq	5′-^YAKYE^ATTGCTCTGGAGTTATGCTATGGGAGTTGGTG^BHQ1^-3′ (1097–1128)
	CDV_N-Rq	5′-GCC TCT TCC TTG GTG ATG-3′ (1279–1262)

^a^Abbreviations: FAM, 6-Carboxyfluorescein; BHQ-1, Black Hole Quencher 1; YAKYE, Yakima Yellow.

^b^Reference genomes GenBank accession numbers: CDV, MF041963.1; BoAstV CH13, NC_024498.1.

### Internal positive control

To control for RNA extraction, RNA degradation and PCR inhibitors, we used an internal control system using canine distemper virus (CDV). CDV specific primers and a Yakima Yellow (4,7,2′-trichloro-7′-phenyl-6-carboxyfluorescein) labeled probe targeting the CDV nucleoprotein (N) coding region were designed (Table 2). Total RNA was extracted from a stock of culture supernatant derived from CDV (strain Onderstepoort) infected Vero cells expressing the canine SLAM receptor (Vero-cSLAM [kindly provided by V. von Messling]) with a TCID_50_ of 3.1 × 10^4^ per ml. RNA extracts were tested by RT-qPCR using the same conditions as described below for the astrovirus RT-qPCRs, but with CDV-specific oligonucleotides (Table 2).

### Synthetic RNA controls

A cDNA sequence corre*s*ponding to the entire BoAstV-CH13 reference genome (GenBank accession number NC_024498.1) was synthesized and cloned by a commercial service (Eurofins) in two fragments: pJET_BoAstV-CH13_Frag A, and pGH_BoAstV-CH13_Frag B. Both fragments were linked by Gibson PCR and cloned into the low copy plasmid pACJR^20^ (kindly provided by N. Ruggli, Institute of Virology and Immunology, Mittelhäusern, Switzerland) resulting in the construct pACJR_BoAstV-CH13-#55. The sequence was flanked by a T7 promoter sequence at the 5′ end and a unique SwaI site at the 3′ end. The selected clone (#55) showed a nucleotide insertion in ORF 1a at position 1334, which leads to a premature stop codon in ORF 1A at position 1387. pACJR_BoAstV-CH13-#55 was linearized with SwaI and run-off RNA transcription was done with the mMESSAGE mMACHINE T7 Transcription kit (Life technologies). The resulting RNA transcripts were quantified with a Fragment Analyzer (Advanced Analytical) and copy numbers were determined with the Endmemo DNA/RNA copy number calculator (http://endmemo.com/bio/dnacopynum.php), based on the RNA peak concentration and the molecular mass of the transcript.

### RNA extraction

Brain tissue samples of ~50 mg were spiked with 10 µl of the CDV stock and were homogenized manually in 1 ml TRI reagent (Sigma-Aldrich) with a 1 ml pipette tip and extracted according to the instructions of the manufacturer. RNA extracts were stored at −80 °C and were thawed and treated on ice only when being further processed.

### RT-qPCR

All RT-qPCR reactions were done with the AgPath-ID One-Step RT-PCR System (New England Biolabs) in 25 µl reactions according to the manufacturer’s instructions. The different astrovirus specific RT-qPCR protocols were each combined in duplex reactions with the CDV RT-qPCR. The final concentration in the reaction mix was 400 nM for each primer, and 120 nM for each probe. One µl of sample RNA extract was added to each reaction. RT-qPCR was performed in MicroAmp Optical 96-well reaction plates (Life technologies) in a 7300 Real-time PCR System (Applied Biosystems) with the following cycle settings: 10 min, 45 °C; 10 min, 95 °C and 40 cycles (15 sec, 95 °C; 20 sec, 62 °C; 30 sec, 60 °C). Fluorescence was measured at the end of each elongation step with the FAM filter for the astrovirus RT-qPCR protocols and with the VIC filter for the CDV RT-qPCR. Data were analyzed with the Sequence Detection Software (Applied Biosystems, Version 1.4) applying automatic baseline detection and a manual threshold of 0.2 for astrovirus and 0.1 for CDV detection, respectively. Linear regression analysis was performed with the GraphPad prism software and amplification efficiencies (*E*) were calculated according to the following formula: $E={10}^{-(\frac{1}{n})}-1$; with *n* corresponding to the slope of the calibration curve. Conventional pan-astrovirus one-step RT-PCR was conducted as described previously^21^.

### Repeatability

Intra-test repeatability was assessed by comparison of duplicate analysis of the same RNA extracts on the same plate and by linear regression using the Prism GraphPad software (Version 5.0). Inter-test repeatability was assessed by testing brainstem tissues of positive (n = 2) and negative (n = 2) tissue homogenates in 20 independent RNA extraction and RT-qPCR runs on different days by 4 different people.

### Sequencing

Sanger sequencing of RT-qPCR amplicons was conducted with the BigDye Terminator sequencing kit (Applied Biosystems) and an ABI 3730 capillary sequencer. Next-generation sequencing was done on brain tissue total RNA extracts in paired-end mode with a length of 150 bp on an Illumina HiSeq 3000, yielding ~200 Mio reads per sample. Reads were then aligned to all viral genome entries from Genbank and RefSeq [accessed 12 April 2018, using bowtie2 (version 2.3.0)]^22^.

## Results

**Figure 1:**
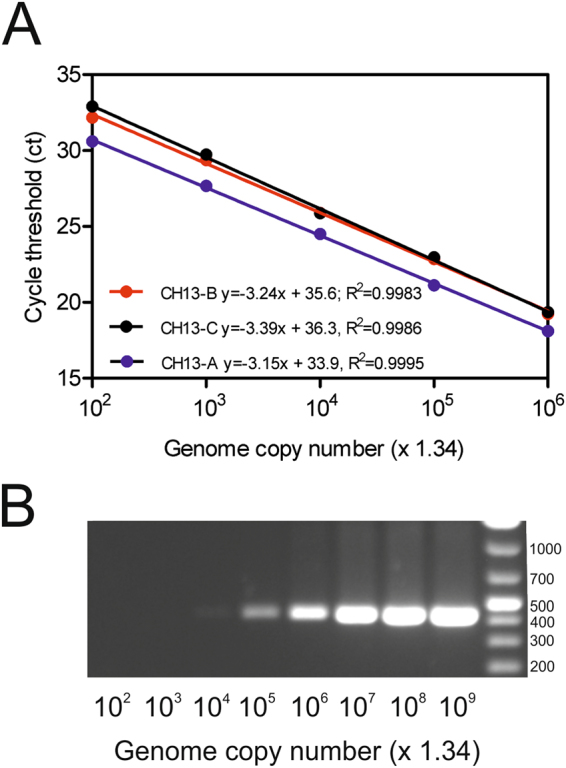
(**A**) Analytical sensitivity of three RT-qPCR assays for bovine astrovirus CH13/NeuroS1 (BoAstV-CH13/NeuroS1). *In* *vitro* transcribed genomic RNA was serially diluted tenfold and tested in the CH13-A, CH13-B and the CH13-C assays. Positive results were obtained for ≥1.34 × 10^2^ RNA copies (cut-off ct ≤35). Calibration curves were generated by linear regression and the resulting linear regression coefficients (*R*^2^) were ≥ 0.99. (**B**) Conventional pan-astrovirus RT-PCR using the same dilution of the BoAstV-CH13/NeuroS1 RNA transcript. Molecular masses of the DNA marker are indicated in bp on the right. The image has been cropped and rotated using the CorelDRAW X6 software. The full-length gel is presented in Fig. S2.

*In* *vitro* transcribed full-length viral RNA from cloned cDNA served for the determination of the limit of detection (LOD) of the three astrovirus RT-qPCRs. The RNA stock showed a concentration of 1.4 × 10^10^ RNA copies per µl. All three assays detected viral RNA in dilutions of up to 1:10^8^, which corresponds to ~134 RNA copies. Cycle threshold (ct) values of these dilutions were between 30.5 and 33 and no RNA was detected in dilutions of 1:10^9^, which results in a LOD between 13.4 and 134 RNA copies. The RT-qPCR amplification efficiencies were 107.5% (CH13-A) 103.6% (CH13-B) and 97.3% (CH13-C), respectively, with a linearity (*R*^2^) > 0.99 (Fig. 1A). Based on these results, the cut-off for astrovirus detection was set at a ct value of 35 for all three RT-qPCR assays. In comparison, the conventional pan-astrovirus one-step RT-PCR had a limit of detection between 1.34 × 10^4^ and 1.34 × 10^3^ copies, and was thus around two orders of magnitude less sensitive than the BoAstV-CH13/NeuroS1 specific RT-qPCR protocols (Fig. 1B).

To monitor the efficiency of the RNA extractions, each tissue sample was spiked with a standardized amount of CDV. Total RNA extracted from 10 µl CDV stock constantly yielded cycle threshold (ct) values of around 30. Similar ct values were observed in total RNA extracts of cattle brain tissue previously spiked with 10 µl of the CDV stock. We therefore decided to spike 10 µl of this CDV stock, which corresponds to 3.1 × 10^2^ TCID_50_, into each ruminant brain tissue sample prior to RNA extraction and to analyze each sample for CDV in a duplex RT-qPCR setup together with each astrovirus protocols. CDV was detected in 48 out of 60 astrovirus-positive and negative spiked tissue samples (mean ct of 30, SD ± 3.5). However, in a total of 12 astrovirus-negative control samples at least one of the duplicates in at least one of the three RT-qPCR assays was CDV-negative. Retesting of these samples following new RNA extraction gave positive CDV results in seven of these samples. In the remaining five we were not able to detect CDV RNA after several extraction trials and these samples were excluded from the validation study. These samples were replaced by other CDV-spiked astrovirus-negative control samples, in which the CDV RNA was detected by RT-qPCR.

**Figure 2:**
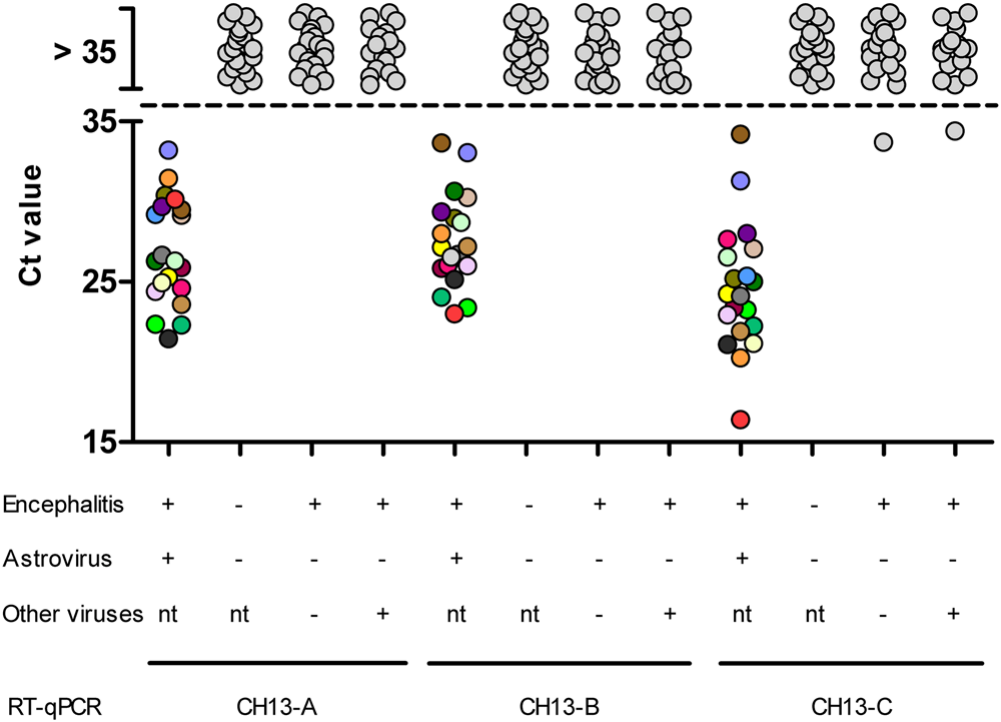
Accuracy of bovine astrovirus CH13/NeuroS1 (BoAstV-CH13/NeuroS1) RT-qPCR assays. The diagnostic sensitivity was determined by testing brain tissue samples with non-suppurative encephalitis and a BoAstV-CH13/NeuroS1 positive status (encephalitis+/astrovirus+) (n = 20), and the diagnostic specificity by testing samples without histopathological brain lesions and with a BoAstV-CH13/NeuroS1 negative status (encephalitis−/astrovirus−) (n = 20). Finally, the analytical specificity was assessed on samples with a non-suppurative lesions pattern, in which no virus was detected (n = 20), and on samples with encephalitis, in which viruses other than BoAstV-CH13/NeuroS1 were identified (n = 17). The positive/negative cut-off at cycle threshold (ct) 35 is indicated by a dashed line. Individual astrovirus positive samples are coded by colors.

All negative control samples of the healthy slaughtered cattle scored negative and all astrovirus-positive samples scored positive in the CH13-A, the CH13-B and the CH13-C RT-qPCRs, which results in a diagnostic specificity of 100% and a diagnostic sensitivity of 100% for all three tests (Fig. 2).

The astrovirus-negative encephalitis group (n = 20) and the RNA extracts of cattle brain tissues with a known infection status (n = 17) served to assess the analytical specificity of the test formats. Two samples gave a positive signal in the CH13-C RT-qPCR, with a ct value of 33.8 (animal ID 47492) and 34.4 (animal ID 47476, BHV-6-positive, Table S2) respectively, while all other samples of these groups scored astrovirus negative in all three assays. Sanger sequencing of the CH13-C amplicons of the two positive samples revealed no significant hit for ID 47492, but a *Bos mutus* (wild yak) chromosome 5 (GenBank: CP027073.1) sequence for ID 47476. Moreover, NGS of the RNA extracts of both samples did not reveal the presence of sequence reads matching to other known viral sequences, including astroviruses. Therefore, both samples are regarded as false positive. Taken together, these results reveal an analytical specificity of 95% for CH13-C and of 100% for CH13-A and CH13-B (Fig. 2) in bovine brain samples.

None of the RNA extracts from the bovine feces samples (n = 6) that were positive for enteric astroviruses and from the astrovirus-negative feces samples (n = 9) was positive in the three RT-qPCR assays, indicating the absence of cross-reactivity with phylogenetically unrelated bovine astroviruses.

**Figure 3:**
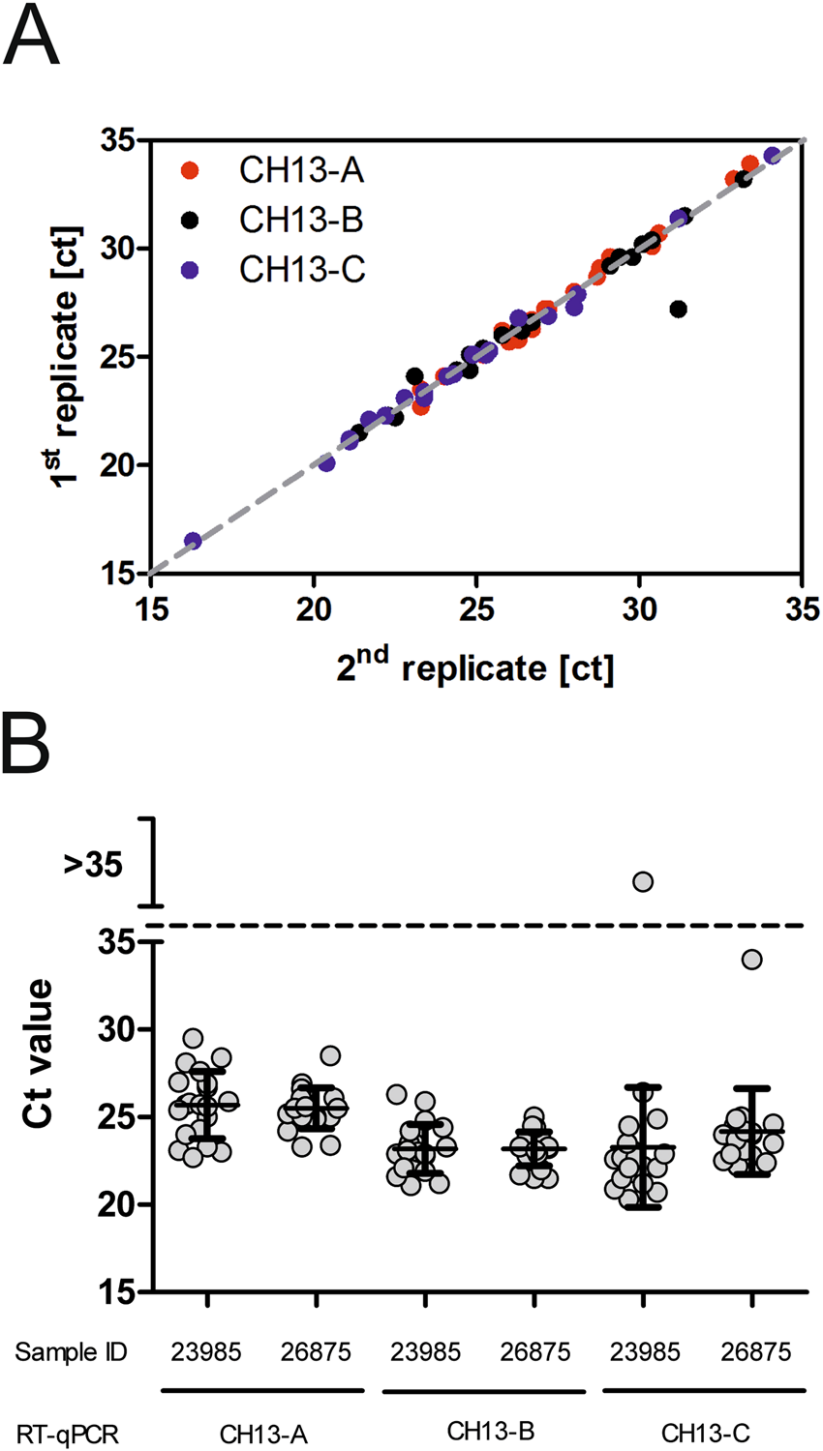
Precision of bovine astrovirus CH13/NeuroS1 (BoAstV-CH13/NeuroS1) RT-qPCR assays. (**A**) Intra-test repeatability of the three assays (CH13-A, CH13-B and CH13-C) was assessed by testing two replicates of 20 different BoAstV- CH13/NeuroS1 tissue RNA extracts in the same test run. (**B**) Inter-test repeatability was determined by testing BoAstV-CH13/NeuroS1 positive (n = 2; 23985 and 26875) and negative tissue homogenates (n = 2) in 20 independent test runs with separate RNA extraction. Negative tissue homogenates tested consistently negative (data not shown). For positive tissue extracts, individual ct values (dots) are presented together with average scores and standard deviations (error bars). The positive/negative cut-off at cycle threshold (ct) 35 is indicated by a dashed line.

Linear regression analysis of duplicate results showed R^2^ values of >0.99 for CH13-A and CH13-C and of >0.92 for CH-B, which indicates an excellent association between test results on the same plate and thus a very good intra-test repeatability (Fig. 3A). The coefficients of variation of ct values of inter-test replicates (n = 20) of two BoAstV-CH13 positive samples was ≤7.5% for CH13-A as well as CH13-B, and in the range of 10–15% for CH13-C (Fig. 3B). The same number of replicates of two BoAstV-CH13 negative samples yielded negative results in all three RT-qPCR assays.

## Discussion

BoAstV-CH13/NeuroS1 has been identified recently as a biologically plausible cause of non-suppurative encephalitis in cattle. Based on our previous molecular characterization of full length BoAstV-CH13/NeuroS1 genomes^23^, we designed and validated three different RT-qPCR protocols for molecular diagnostics of this virus. Primers and probes were selected according to best matches to the consensus sequence of 11 whole genome sequences of BoAstV-CH13/NeuroS1 isolates. These strains (i) covered different geographical origins, i.e. Switzerland and the USA, (ii) were derived from tissue samples that had been collected over a time span of more than 30 years and (iii) were characterized by a broad range of molecular and immunochemical techniques. Thus, they are expected to be representative for the spectrum of genetic variants within the encephalitis-associated BoAstV-CH13/NeuroS1 genotype species.

While the target sequences for the primers and probes of the RT-qPCR assay CH13-A were fully conserved in all isolates, this was not the case for those of assays CH13-B and CH13-C. The forward primer BoAstV_qL of CH13-B and the reverse primer CH13_6450Rq of CH13-C showed mismatches near their 5′ end compared to the sequences of the US NeuroS1 isolate (Fig. S1). Unfortunately, we could not test original brain tissues of the US NeuroS1 isolate, and it therefore remains unknown to which extent it would be detected by these two assays. Moreover, CH13_6339Fq of CH13-C contained a mismatch compared to one of the Swiss strains, ID 26875, at the second position at its 3′ end (Fig. S1). This variant of the viral sequence was reviewed by NGS^23^ and confirmed in >98.5% of the reads (read depth 26′694×). Against expectations, the mismatch does not seem to influence the detectability of this particular strain as it was readily detected in the CH13-C assay with ct values ~24, which is very similar to the isolates that do not show this mismatch.

It is important to mention that the three assays described here were not designed to detect bovins astrovirus CH15 (BoAstV-CH15), an astrovirus strain similarly identified in two cattle with encephalitis in Switzerland^8^ and in Germany (the latter strain was termed BoAstV-BH19/14)^7^. BoAstV-CH15 shares only ~60% identity on nucleotide level with BoAstV-CH13/NeuroS1. None of the assays under investigation in the present study detected BoAstV-CH15 in the Swiss cases (data not shown) and our attempts to identify primer probe combinations suitable for BoAstV-CH13/NeuroS1 and BoAstV-CH15 detection were not successful. For this strain, different primer-probe combinations need to be established. However, these could potentially be combined with one or more of the BoAstV-CH13/NeuoS1 protocols in a multiplexed assay format.

The LODs and amplification efficiencies for BoAstV-CH13/NeuroS1 were similar for the three assays and superior to a previously described conventional RT-PCR. Analysis of brain tissue samples of BoAstV-CH13/NeuroS1 positive cattle and of negative healthy cattle resulted in a diagnostic sensitivity and diagnostic specificity of 100% for all three assays. For the majority of the positive tissue samples, the ct values were clearly below the defined cut-off of 35, which demonstrates a high discriminatory power.

Of the BoAstV-CH13/NeuroS1 negative animals with non-suppurative encephalitis, two animals were positive in the CH13-C RT-qPCR with relative high ct values >33, but negative in the two other assays. These animals were tested astrovirus negative by conventional pan astrovirus RT-PCR and NGS. Because sequencing of the CH13-C amplicon in these cases found astrovirus unrelated sequences, the CH13-C results need to be interpreted as false positive, which reduces the analytical sensitivity of this assay.

The intra- and inter-test repeatability of the three assays were assessed by comparing duplicates of positive samples on the same PCR plate, and by analyzing replicates of individual positive and negative tissue homogenate extracts in different test runs. Both setups yielded excellent results for CH13-A and CH13-B, while the CH13-C assay showed a higher inter-test variation with single outlier test results, and one replicate test run scoring negative. Overall, the variation appeared to be higher between test runs than within the same test run, which suggest that RNA extraction is a source of assay variability.

The performance of the internal CDV control in monitoring RNA extraction and the presence of PCR inhibitors was only partially convincing. In some brain tissue samples the internal CDV control was not detected. Also in the inter-test repeatability trials the RT-qPCR results for CDV were highly variable and remained negative in ~1/3 of all tests (data not shown). Failure of CDV detection may be caused by rapid CDV RNA degradation in the brain tissue matrix. CDV is an enveloped virus with a relatively low tenacity, while astroviruses are non-enveloped and highly resistant to inactivation and degradation. An unrelated internal astrovirus control, e.g. a human astrovirus harvested from infected cell cultures, may be better suited to monitor RNA extraction and RT-qPCR performance of the BoAstV-CH13/NeuroS1 assays.

The rationale to design RT-qPCR protocols specifically targeting the ORF2 was based on the fact that during viral RNA replication, in addition to genomic full-length RNA, subgenomic ORF2-encoding RNA is generated. We expected that this would increase the sensitivity of virus detection. Yet, we found no evidence for the ORF2-aimed assays being more sensitive than the ORF1a assay in the brain tissues under investigation. It is possible that subgenomic RNA was degraded post mortem and that the assays primarily detected particle-encapsidated genomic RNA, which is better protected from degradation. The situation will likely be different in research settings, e.g. for the analyses of cell culture RNA extracts, or for freshly collected samples from infected animals. In that context, this set of RT-qPCRs could assist in dissecting RNA replication on the genomic and subgenomic level.

Overall, the analytical sensitivity, the accuracy and the precision of all assays were very similar; however, most consistent results were obtained with the CH13-A assay, which we suggest for routine diagnostic purposes on brain tissues.

## Electronic supplementary material

Supplementary information
