# Supplementary material for: Accurate and precise real-time RT-PCR assays for the identification of astrovirus associated encephalitis in cattle
Source: Sci Rep. 2018 Jun 15;8:9215. doi: 10.1038/s41598-018-27533-8 (PMC6003944; doi:10.1038/s41598-018-27533-8)
Supplement: Supplementary file 1 — Supplementary information [file 41598_2018_27533_MOESM1_ESM.pdf]

Supplementary information

Accurate and precise real-time RT-PCR assays for the identification of astrovirus associated encephalitis in cattle

Ramona Lüthi[1], Céline L. Boujon[1,2], Ronja Kauer[1], Michel C. Koch[1,2], Ilias G. Bouzalas[1#], and Torsten Seuberlich[1]

[1]NeuroCenter, Division of Neurological Sciences, Vetsuisse Faculty, University of Bern, Bern, Switzerland

[2]Graduate School for Cellular and Biomedical Sciences, University of Bern, Bern, Switzerland

[#]Current address: Veterinary Research Institute, Hellenic Agricultural Organization-DEMETER, Campus of Thermi, Thessaloniki, Greece

Supplementary Figure 1: Primer design for Bovine astrovirus CH13/NeuroS1 (BoAstV-CH13/NeuroS1) RT-qPCR assays. (A) Schematic representation of the BoAstV-CH13/NeuroS1 genome with three open reading frames (ORF1A, ORF1B and ORF2). Target regions for the three RT-qPCR assays (CH13-A, CH13-B and CH13-C are indicated by black bars. (B) Primer and probe binding sites in 11 virus strains for which full-genome sequences are available. Genbank accession numbers are indicated on the left. Polymorphisms are indicated by colored nucleotides and the consensus sequence is shown on the top. Primer and probe binding sites are indicated by green and red arrows, respectively. Alignments were generated with the Geneious Software package (Biomatters, version 9.1.8).

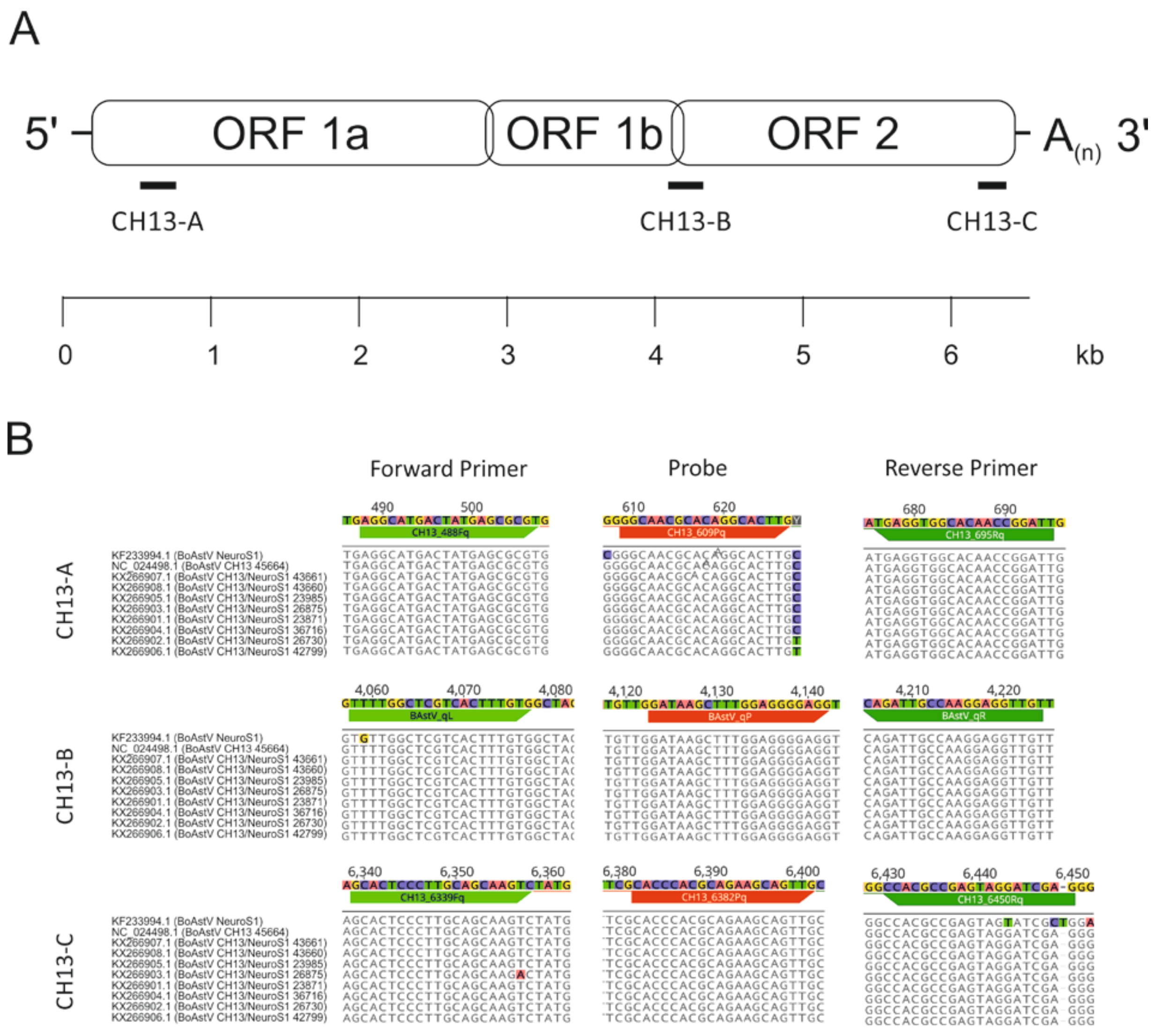

Supplementary Figure 2: Unprocessed gel picture of Figure 2B. The dashed white box indicates the cropped area.

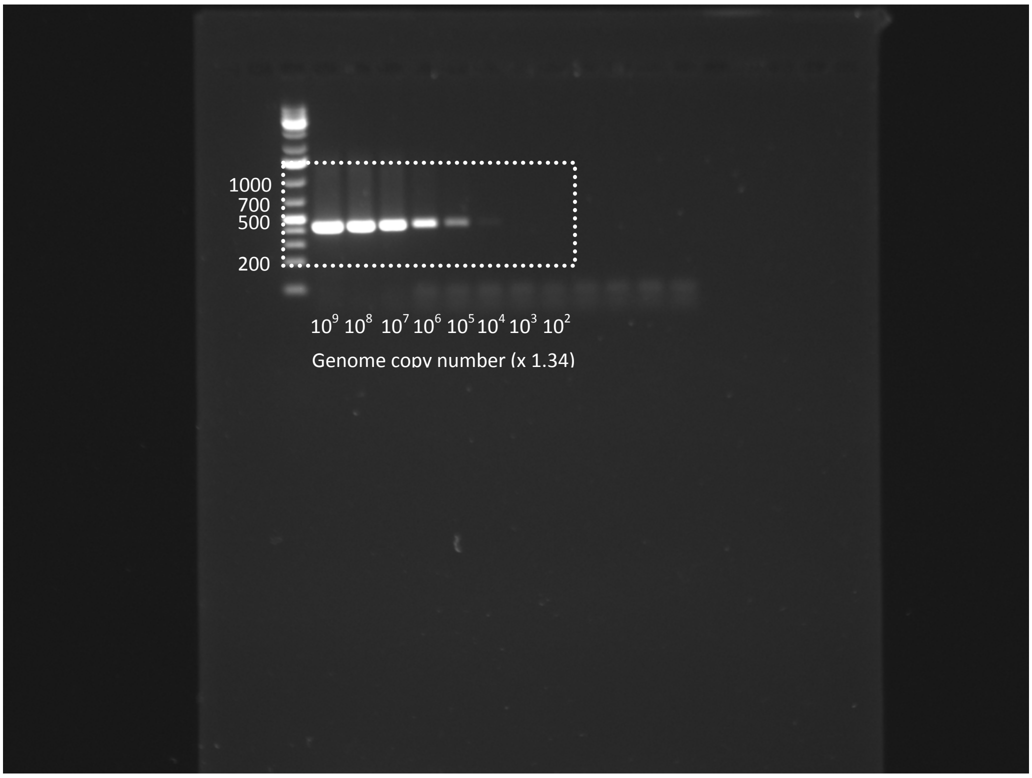

Supplementary Table 1: Details on RNA extracts of cattle brain tissues tested positive for viruses other than BoAstV CH13/NeuroS1: BHV-6, Bovine herpesvirus 6; BoPyV-2, Bovine polyomavirus 2; BoRV Ch15, Bovine retrovirus CH15; OvHV-2, Ovine herpesvirus 2; PIV-5, Parainfluenza virus 5; BVD, Bovine viral diarrhea virus.

**Test format**	**PCR / RT-PCR***					**NGS**	**BoAstV CH13/NeuroS1 RT-qPCR [ct]**		
**Animal ID**	**BHV-6**	**BoPyV-2**	**BoRV CH15**	**OvHV-2**	**PIV-5**	**BVD**	**CH13-A**	**CH13-B**	**CH13-C**
24250	+						nd	nd	nd
25018			+				nd	nd	nd
26324				+			nd	nd	nd
26731	+	+			+		nd	nd	nd
32450	+	+					nd	nd	nd
34510		+					nd	nd	nd
42268	+						nd	nd	nd
47243				+			nd	nd	nd
47417			+				nd	nd	nd
47422				+			nd	nd	nd
47476	+	-					nd	nd	34,4
47681	+						nd	nd	nd
47791	+						nd	nd	nd
48304				+			nd	nd	nd
439						+	nd	nd	nd

+, positive; - negative; nd, not detected; * result have been published previously by Wüthrich et al. [1] and Truchet et al. [2]

Supplementary Table 2: Bovine feces samples tested positive for astrovirus using the pan-astrovirus RT-PCR targeting the viral RNA-dependent RNA polymerase (RdRp) encoded by ORF1b.[3] Amplicons were Sanger sequenced and the resulting sequences were compared with GenBank nucleotide database entries using megablast. Best hits and sequence identities are shown.

Sample ID	Best megablast hit (accession number)	Identity [%]
16D3810	Bovine astrovirus strain BAstGX-J27 (KJ476832)	87.8
16D3962	Yak astrovirus isolate S8 (KM822593)	84.5
17D43	Bovine astrovirus B18/HK, complete genome (HQ916313)	90.0
17D99	Bovine astrovirus strain BAstGX-J27 (KJ476832)	88.9
17D824	Astrovirus deer/CcAstV-2/DNK/2010 (HM447046)	86.2
17D1196	Bovine astrovirus strain BAstGX-J27 (KJ476832)	88.5

References

1 Wuthrich, D. *et al.* Exploring the virome of cattle with non-suppurative encephalitis of unknown etiology by metagenomics. *Virology* **493**, 22-30, doi:10.1016/j.virol.2016.03.009 (2016).

2 Truchet, L. *et al.* Neuropathological survey reveals underestimation of the prevalence of neuroinfectious diseases in cattle in Switzerland. *Veterinary microbiology* **208**, 137-145, doi:http://dx.doi.org/10.1016/j.vetmic.2017.07.027 (2017).

3 Mittelholzer, C., Hedlund, K. O., Englund, L., Dietz, H. H. & Svensson, L. Molecular characterization of a novel astrovirus associated with disease in mink. *Journal of general virology* **84**, 3087-3094 (2003).
